# Delivery of epirubicin via slow infusion as a strategy to mitigate chemotherapy-induced cardiotoxicity

**DOI:** 10.1371/journal.pone.0188025

**Published:** 2017-11-13

**Authors:** Fang Yang, Qiao Lei, Lu Li, Jian Chang He, Jiajia Zeng, Chunxiang Luo, Sai-Ching Jim Yeung, Runxiang Yang

**Affiliations:** 1 The Second Department of Medical Oncology, The Third Affiliated Hospital of Kunming Medical University, Kunming, Yunnan, The People’s Republic of China; 2 The Second Department of Medical Oncology, Yunnan Tumor Hospital, Kunming, Yunnan, The People’s Republic of China; 3 Queen Mary College of Medicine, Nanchang University, Nanchang, Jiangxi, The People’s Republic of China; 4 Department of Pharmacy, Kunming General Hospital of Chengdu Military Command, Kunming, Yunnan, The People’s Republic of China; 5 Department of Emergency Medicine & Department of Endocrine Neoplasia and Hormonal Disorders, The University of Texas MD Anderson Cancer Center, Houston, Texas, United States of Ameirca; Universita degli Studi di Parma, ITALY

## Abstract

**Background:**

Continuous infusion of doxorubicin has been a strategy to reduce cardiotoxicity. Epirubicin is another anthracycline in common clinical use. However, evidence is lacking regarding whether this strategy can reduce cardiotoxicity of epirubicin without compromising antineoplastic efficacy.

**Design and methods:**

Healthy rats were randomized into groups: epirubicin (8 mg/kg) delivered intraperitoneally via micro osmotic pumps (MOP), epirubicin (8 mg/kg) by intraperitoneal (IP) bolus injection, and placebo control. Blood samples were collected for analyzing biomarkers of myocardial injury and pharmacokinetics. At chosen times, sub-groups of animals were sacrificed for histopathology. A mouse breast cancer cell line (4T1), stably transfected with luciferase, was orthotopically allografted in female mice, and treated in three groups as described above for the rats. Tumor growth was monitored by measuring tumor size as well as bioluminescence.

**Results:**

Delivery by IP bolus and by MOP achieved essentially the same area under the curve of epirubicin plasma concentration time profile. Blood biomarkers showed that the degree of myocardial injury in MOP group was lower than that of IP group. Histopathology showed that there was less eosinophilic enhancement, interstitial hemorrhage and necrotizing muscle atrophy in MOP group than IP group. In the orthotopic breast cancer allograft mouse model, the antineoplastic effect of epirubicin by MOP was not different from that by IP as measured by tumor weights or by *in vivo* bioluminescence.

**Conclusion:**

Slow delivery of epirubicin by MOP reduced cardiotoxicity without compromising the antineoplastic effect compared to IP bolus delivery. These *in vivo* data support our previous clinical data that continuous intravenous infusion of epirubicin using micro infusion pumps over 48–96 hours had less cardiotoxicity than intravenous bolus injections. However, whether multiple doses of epirubicin given by MOP result in a lower magnitude of long term cardiomyopathy remains to be further investigated.

## Introduction

Anthracyclines are effective antineoplastic agents against malignancies such as breast cancer, lymphoma, lung cancer, ovarian cancer, sarcomas, liver cancer, and etc. However, the cardiotoxicity of anthracycline-based chemotherapy is cumulative, and it sets limits on the life-time cumulative dose of anthracyclines that can be safely administered to cancer patients, thus limiting the therapeutic options for a large number of patients with advanced disease [1]. The toxic mechanism of anthracyclines is still unclear. As free radicals may be important mediators of anthracycline-induced cardiotoxicity [2], chelation of iron by anthracycline-based drugs may be the primary cause that generate oxygen free radicals, leading to myocardial cell membrane lipid peroxidation and myocardial mitochondrial DNA damage [3].

Several strategies have been used to reduce cardiotoxicity of anthracycline-based chemotherapy [4, 5]: first, by choosing anthracycline derivatives with less cardiotoxicity (e.g., epirubicin); second, by co-administration of drugs with a cardioprotective effect (e.g., dexrazoxane); third, by novel formulations of anthracyclines (e.g., PEGylated liposomal doxorubicin) [6]; and fourth, by continuous infusion [7]. Continuous infusion of epirubicin (5 mg/m^2^/day) for 21 days has been studied perhaps as a metronomic dosing regimen [8–12]. Similar to what had been tried for doxorubicin [7], a 4-day infusion schedule was investigated for leukemia [13]. We previously studied 48-hour infusion of epirubicin (70 mg/m^2^ in total) [14–16] with the goal to reduce the cardiotoxicity of epirubicin in mind, and we found that continuous intravenous infusion of epirubicin using micro-pumps had less cardiotoxicity than intravenous bolus infusion of epirubicin at the same total dose of 70 mg/m^2^ [14, 17].

Yet direct histopathological evidence for reduction of cardiotoxicity by changing the method of epirubicin administration is lacking. More importantly, the *in vivo* antineoplastic efficacy of bolus needs to be compared with slow continuous delivery. Therefore, we performed rodent studies to compare the cardiotoxicity of epirubicin administered as intraperitoneal bolus injections and that of slow infusion into the peritoneal cavity over 72 hours using mini-osmotic pumps. A breast cancer mouse model was used to compare the antineoplastic efficacies of these two methods of epirubicin delivery. The difference in pharmacokinetics of the two delivery methods was also examined as a potential explanation for any observed differences in cardiotoxicity. Our result will lay an experimental basis to use continuous infusion of epirubicin over 2–3 days as the preferred method of administration over intravenous bolus infusions for the sake of cardioprotection in cancer patients.

## Materials and methods

### Materials

Pharmaceutical grade epirubicin hydrochloride for injection (10 mg/vial) was purchased from Zhejiang Haizheng Pharmaceutical Co., Ltd. (Taizhou, Zhejiang, PRC). Ketamine hydrochloride injection (100 mg/vial) was purchased from Fujian Gutian Pharmaceutical Co., Ltd. (Gutian, Fujian, PRC). D-Luciferin was purchased from Beijing Pan-Bo Biochemical Co., Beijing, PRC). Alzet micro-osmotic pumps (Model: 1003D) was produced by DURECT Corporation (Cupertino, CA).

### Cell culture

The mouse breast cancer cell line, 4T1, was stably transfected with a plasmid expressing luciferase and puromycin resistance gene (4T1-luc, originally referred to as 4T1-luc2 by Qin et al. in their publication) [18]. 4T1-luc cells were cultured in RPMI-1640 medium (HyClone Cell Culture Products, GE Healthcare Bio-Sciences, Pittsburgh, PA) supplemented with 10% fetal bovine serum (FBS) (HyClone) and 0.01% of puromycin (InvivoGen, San Diego, CA). The cells were cultured in a humidified atmosphere with 5% CO_2_ at 37°C, and were passaged every 3−5 days.

### Drug sensitivity testing *in vitro*

Cultured cells in log-phase of growth were incubated for 48 hours in control medium or in various concentrations of epirubicin in 96-well plates. The total biomass of the cells was measured using the sulforhodamine B (SRB) assay according to standard procedures. Optical density at 530 nm (OD_530_) of each well was measured in order to calculate the inhibition rate relative to placebo control.

### Animals

Animal experiments were conducted under approved protocols of Kunming Medical University. All applicable international, national, and institutional guidelines for the care and use of animals were followed. All procedures performed involving animals were in compliance with the ethical standards of Kunming Medical University and Yunnan Tumor Hospital.

Toxicity and Pharmacokinetic Experiments: Male Sprague-Dawley rats were purchased from Kunming Medical University Animal Laboratory. The rats were 6−9 weeks old, and weighed 180−220 g.

Therapeutic Efficacy Experiments: An orthotopic allograft breast cancer mouse model was used. Female Balb/c mice were purchased from Hunan SJA Laboratory Animals, LLC (Changsha, Hunan, PRC). The mice were 5−6 weeks old, and weighed 18−20 g. The 4T1-luc cells were harvested at the logarithmic growth phase. Each mouse was inoculated with 0.1 mL of cell suspension containing 1×10^5^ cells at the right third or fourth mammary fat pads. In about one week after inoculation when the diameter of breast cancer reached 3−5 mm, the mice were randomized into treatment or control groups.

### Toxicity evaluation

One hundred fifty healthy rats were randomly assigned into three groups: 54 rats received intraperitoneal epirubicin injection (control treatment group), 84 rats received epirubicin via micro osmotic pumps (experimental treatment group), and 12 rats received no treatment (no treatment group). Both control and experimental treatment groups received 8 mg/kg body weight of epirubicin in each rat. For the control treatment group, 6 rats were randomly selected for sacrifice at 2h, 4h, 8h, 12h, 24h, 36h, 48h, 60h, and 72h after intraperitoneal injection of epirubicin. For the experimental treatment group, pharmaceutical preparation of epirubicin was reconstituted in sterile water for injection to the appropriate volume for pump loading, and each micro osmotic pump was intraperitoneally. After surgical closure of wounds and recovery, 6 rats were randomly selected for sacrifice at 12h, 24h, 36h, 48h, 60h, 72h, 76h, 80h, 84h, 96h, 108h, 120h, 132h, and 144h after pump implantation. For the no treatment group, 3 rats were randomly selected for sacrifice at 24h, 48h, 72h, and 96h after group assignment.

At sacrifice, each animal was anesthetized with ketamine and then the heart is exposed after thoracotomy. Venous blood was collected by R atrial puncture. A minimum of 6 mL of blood was collected, and used for blood cell analysis (1.5 mL) and biochemical evaluation for myocardial injury (3 mL) and pharmacokinetics (1 mL).

Each heart was then cut open along the long axis and then fixed by 10% formalin. The fixed tissue was prepared for sectioning and staining with hematoxylin and eosin (H&E) staining according to standard procedures. Histopathological analysis include examination for pathology changes such as the cavitational degeneration of myocardium, myocardial edema, myofibril fracture and dissolution, myocardial dot hemorrhage, myocardial dot necrosis, and interstitial fibrosis. Independently, a researcher blinded to treatment assignment examined and scored the degree of myocardial damage according to the method of Bertazzoli et al. [19].

An Automatic Biochemistry Analyzer (Beckman Coulter Model Au2700) was used to measure the following in plasma samples: Troponin I, creatine phosphokinase (CK), creatine phosphokinase isoenzyme MB (CK-MB), α-hydroxybutyric acid dehydrogenase, and lactate dehydrogenase. A Hematology Analyzer (Mindray BC-5100) was used to perform complete blood counts: leukocytes, neutrophils, erythrocytes, and platelets.

### Pharmacokinetic detection and analysis

Blood samples of approximately 1 mL from intraperitoneal injection group and epirubicin micro osmotic pump group respectively were centrifuged at 3500 x g for 5 min, and epirubicin concentration in each 200-μl plasma sample was extracted by mixing an equal volume of 1 M sodium bicarbonate and 3.5 ml of ethyl acetate. After collecting the organic phase, ethyl acetate was evaporated at 40°C in dry nitrogen gas. The organic extract was redissolved in 200 μl ethyl acetate, and the amount of epirubicin was measured using liquid chromatography-mass spectrometry (LC-MS, Agilent 1200SL-6410B, Agilent Technologies, Santa Clara, CA) with an Eclipse XDB-C18 LC column (Agilent Tech.).

Pharmacokinetic analysis was performed using R language software packages "PK" and “pkr". Area under the curve (AUC) of the plasma epirubicin concentration time profile was compared between different drug delivery methods using a two-sided t test.

### Therapeutic efficacy of epirubicin in a breast cancer mouse model

The orthotopic allograft breast cancer mouse model was used. Mice bearing tumors measuring 3−5 mm in diameter were randomized into 3 groups: 1) intraperitoneal epirubicin (8 mg/kg body weight) injection every 6 days (control treatment), 2) epirubicin via micro osmotic pumps at the same dose (8 mg/kg body weight) every 6 days (experimental treatment), and 3) no treatment. Tumor volume was measured with a Vernier caliper once every 3 days, and the volume was calculated by ((*a*×*b*^2^)/2) where *a* was the maximum dimension and *b* was the dimension perpendicular to *a*. After 2 cycles of dosing, each mouse was injected intraperitoneally with D-luciferin (30mg/mL, 200μl/mouse), and then bioluminescence imaging (IVIS Lumina XR, Caliper Life Sciences, Inc., Waltham, MA) was performed at 5, 10, and 15 min post injection.

### Statistical analysis

All experiments were repeated ≥3 times. Statistical analysis was performed using Statistical Package for the Social Sciences (SPSS) version 17.0 (IBM Analytics, USA). All measurement data were subjected to normality test and homogeneity of variance test. The results were expressed as the mean ± standard error of the mean (SEM). Student’s t test or nonparametric rank sum test was used to compare two groups where appropriate. One way analysis of variance (ANOVA) was used to analyze more than 3 or more groups with post-hoc intergroup comparisons. Tumor growth was analyzed by repeated measures ANOVA. Proportions were analyzed by Chi square test or Fisher exact test where appropriate.

## Results

### Comparison of pharmacokinetics between intraperitoneal bolus injection and micro osmotic pump infusion

**Fig 1:**
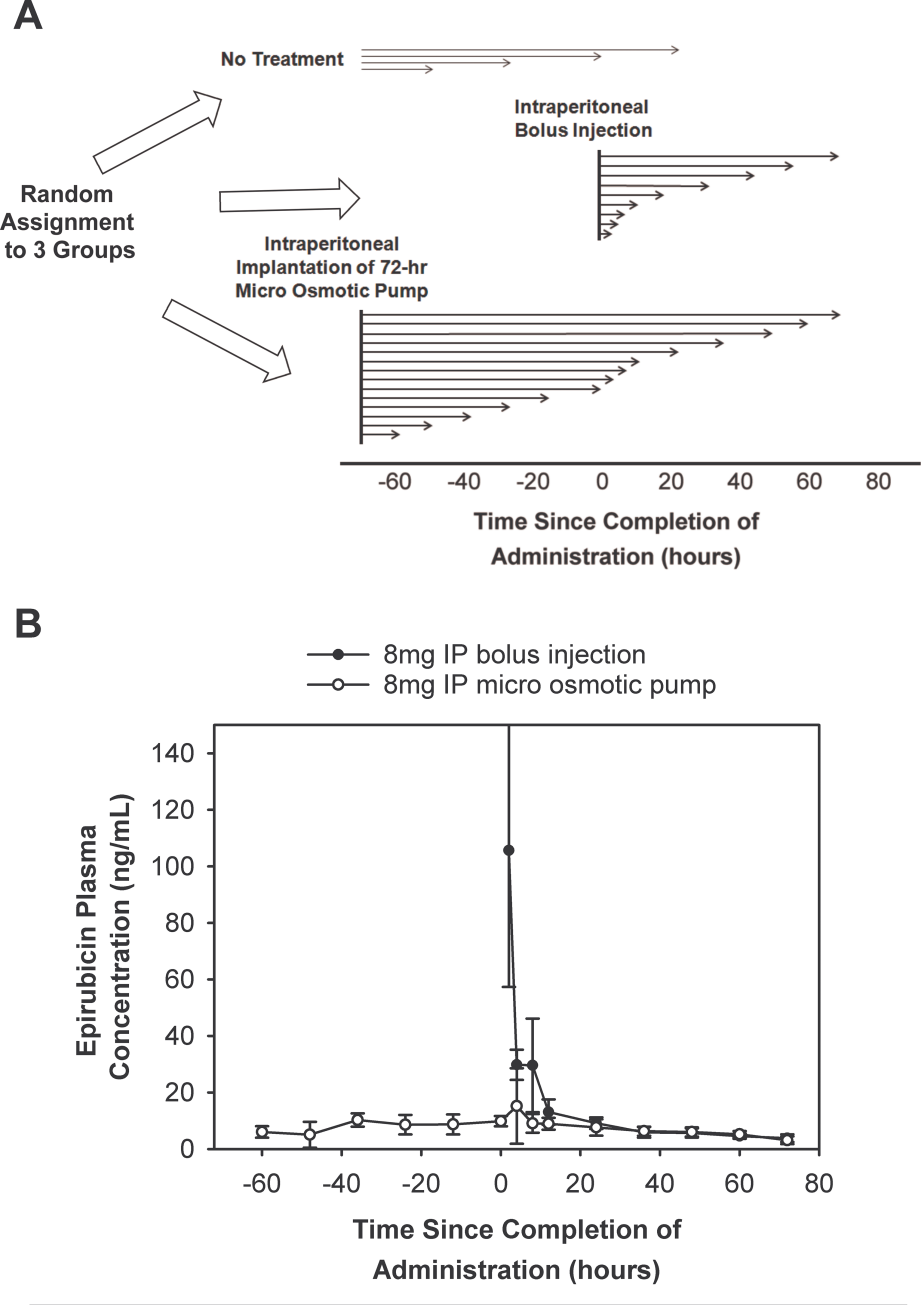
Pharmacokinetic profiles of intraperitoneal bolus injection and intraperitoneal micro osmotic pump infusion in rats. (A) Schematic representation of the experimental design and timing of sample collection. (B) The mean plasma concentrations of the bolus group (solid black circle) and those of the pump infusion group (open circle) are as labeled. The error bars represent the 95% confidence intervals.

From the blood samples collected from the epirubicin cardiotoxicity study in rats described in the Methods section and schematically represented in Fig 1A, the epirubicin pharmacokinetic profile of intraperitoneal bolus injection and that of micro osmotic pump infusion were determined (Fig 1B) and analyzed using non-compartmental analysis (R package “pkr”). The mean dose normalized peak plasma epirubicin concentration (Cmax per mg epirubicin) in the bolus injection group was 14.42 ± 4.99 (s.d.) ng/mL while that in the infusion group was under 2.07 ± 0.27 ng/mL. The plasma concentration of epirubicin was followed for 72 h after completion of infusion (i.e., 144 h after initiation of administration). The area under the curve (AUC) of the bolus injection was 1179.64 ± 407.31 (s.d.) ng-h/mL while that of the infusion was 1142.17 ± 93.23 ng-h/mL. The statistical power of the t test comparison between AUC of bolus injection and AUC of pump infusion was 88.9%. These results showed that bolus injection produced a higher peak plasma epirubicin concentration than pump infusion but their AUCs were equivalent. These data provided the context for the interpretation of histopathological and biochemical evidence of epirubicin cardiotoxicity.

### Histopathological evidence of a higher degree of myocardial damage by intraperitoneal bolus injection than pump infusion of epirubicin

**Fig 2:**
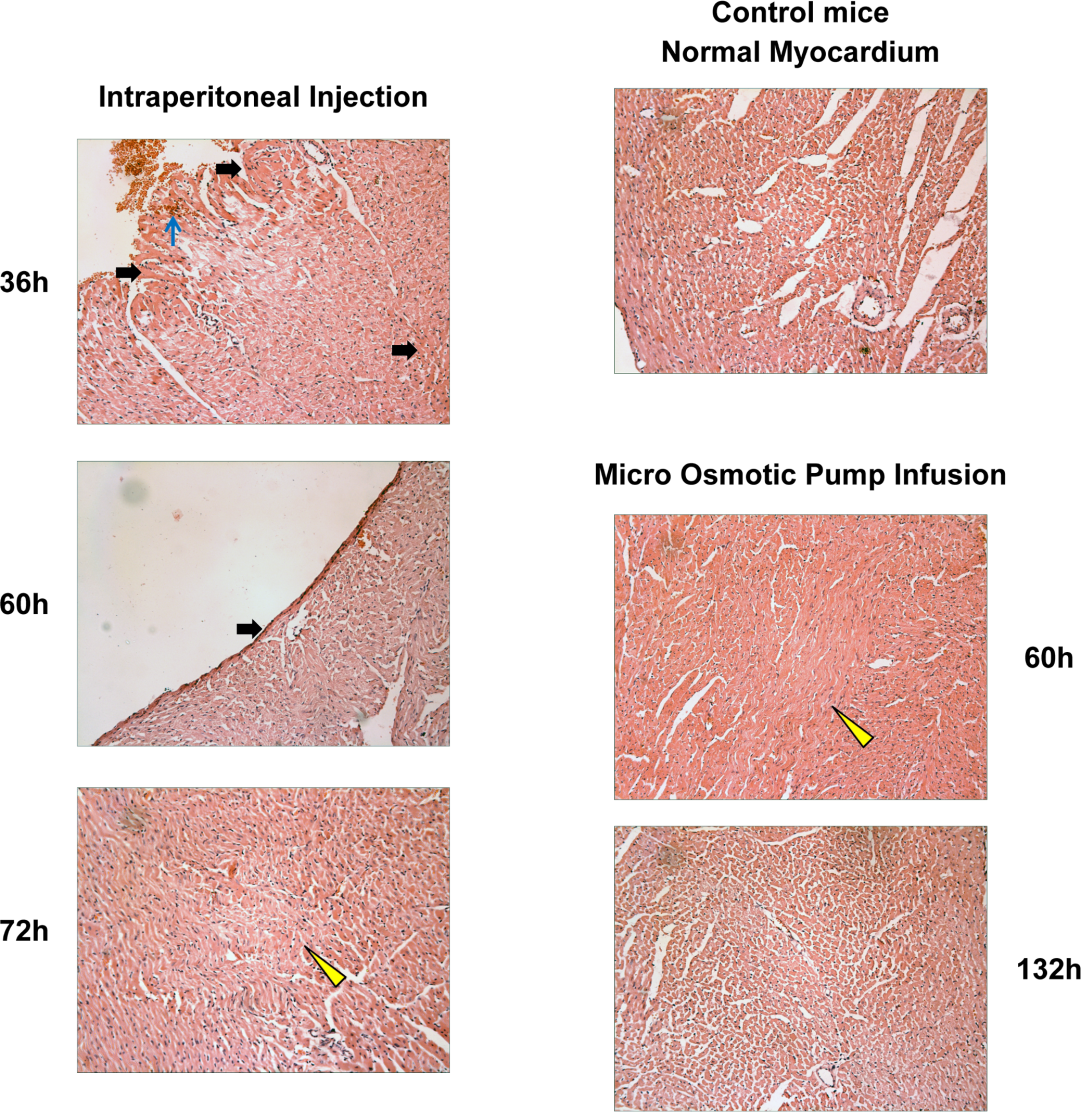
Histopathology of epirubicin-induced myocardial damage. Representative photomicrographs of hematoxylin-eosin-stained slides of rat heart tissues from 3 groups of mice [control, epirubicin (bolus), and epirubicin (infusion)] are shown as labeled. Features such as focal interstitial hemorrhage (blue arrow), eosinophilic enhancement (solid black arrow), and wavy myocardial fibers (yellow arrow head) are indicated. Due to heart beats, paralyzed myocardial fibers become wavy, and this is a sign of loss of contractility.

Myocardial interstitial hemorrhage, increased eosinophilia and even myocardial cell vacuoles degeneration, pyknosis and dissolved nuclei can both be observed in epirubicin micro osmotic pump group and intraperitoneal injection group. The most serious damage appeared at 36−48h after intraperitoneal injection (Table 1). There was detectable injury after the first day of pump insertion, but there was no obvious peak time of myocardial injury. There was increased eosinophilia in a significantly (Fisher Exact test, P<0.05) higher proportion of animals in the intraperitoneal bolus group than in the pump infusion group at 36, 48, 60 and 72h after completion of epirubicin delivery. There was interstitial hemorrhage in a significantly (P = 0.015) higher proportion of animals in the intraperitoneal bolus group than in the pump infusion group at 36 h. There was necrotizing myocardial atrophy in a significantly (P = 0.015) higher proportion of animals in the intraperitoneal bolus group than in the pump infusion group at 48 h. Comparisons of the Bertazzoli score, a composite score of histopathological myocardial damage, showed that the score in the bolus injection group was significantly (P<0.0056, Mann-Whitney test with Bonferroni correction for multiple testing) higher than the pump infusion group at 24 to 48 hours after completion of epirubicin delivery (Table 1). The peak mean Bertazzoli score for the intraperitoneal bolus group was 8, and that for the pump infusion group was 2.83. While typical histopathological evidence of myocardial damage was seen in myocardial samples after bolus injection of epirubicin, few changes were observed in the pump infusion group (Fig 2).

**Table 1:** Comparisons of histopathological features of myocardial damage.

	Eosinophilic enhancement*			Interstitial hemorrhage*			Necrotizing muscle atrophy*			Bertazzoli Score^#^		
	IP bolus	Pump	Exact test	IP bolus	Pump	Exact test	IP bolus	Pump	Exact test	IP bolus	Pump	U test
			P value			P value			P value			P value
Initiation												
12h		0/6			3/6			0/6			0	
24h		1/6			1/6			0/6			0.083	
36h		1/6			1/6			0/6			0.667	
48h		1/6			3/6			0/6			0.667	
60h		0/6			0/6			1/6			1.333	
72h		0/6			1/6			0/6			0.5	
Hours post												
2h	0/6			0/6			0/6			0		
4h	0/6	0/6		0/6	3/6	0.182	0/6	0/6		0	0.6667	1
8h	1/6	1/6	1	2/6	1/6	1	0/6	0/6	1	0.417	0	0.394
12h	1/6	0/6	1	4/6	1/6	0.242	2/6	0/6	1	1.33	0.1666	0.818
24h	2/6	1/6	1	6/6	3/6	0.182	2/6	2/6	1	3.75	2.8333	0.004
36h	6/6	1/6	0.015	5/6	0/6	0.015	1/6	0/6	1	8	0.1667	0.002
48h	5/6	0/6	0.015	1/6	1/6	1	5/6	0/6	0.015	6.667	0	0.002
60h	5/6	0/6	0.015	2/6	0/6	0.455	0/6	0/6	1	3.667	0	0.015
72h	5/6	0/6	0.015	3/6	0/6	0.182	0/6	0/6	1	2.667	0	0.015

* Reported as the number of animals with the histopathological feature detected over the number of animals examined; e.g., 2/6 means two animals had the feature out of 6 animals examined.

^#^ Reported as the mean score of 6 animals examined.

### Biochemical evidence of a higher degree of myocardial damage by intraperitoneal bolus injection than pump infusion of epirubicin

**Fig 3:**
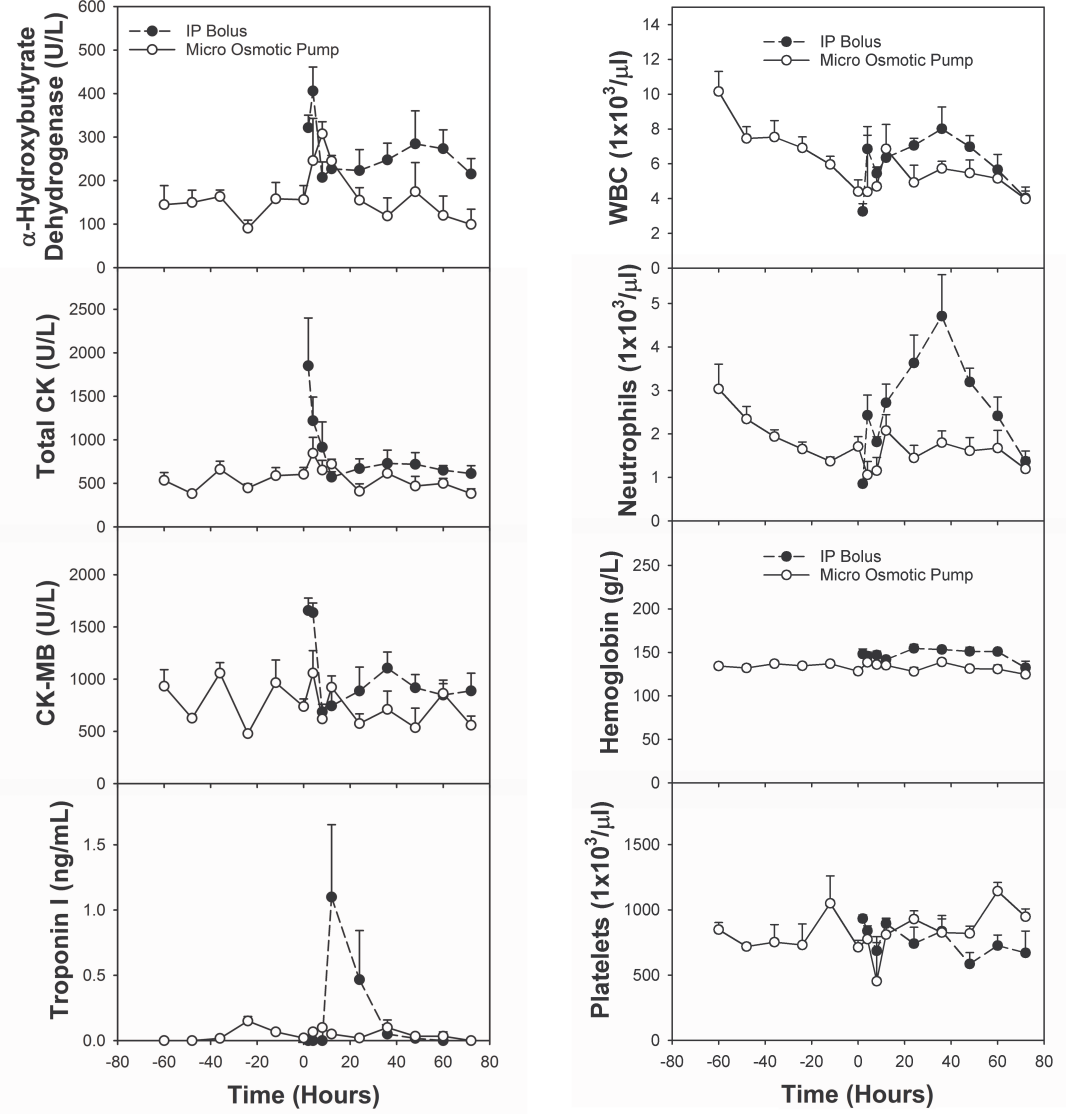
Differential impact of two epirubicin administration methods on biomarkers of myocardial damage and complete blood counts. The IP bolus group is represented by solid black dots, and the micro osmotic pump group by white dots. The horizontal axis is the time since completing epirubicin administration in hours. Error bars represent standard error of the mean.

Plasma biomarkers of myocardial injury [α-hydroxybutyrate dehydrogenase, creatine phosphokinase (total CK), creatine phosphokinase-MB fraction (CK-MB), and troponin-I] were measured from blood samples collected from rats treated as reported above and as described in Methods. The average values were plotted against time with time 0 defined as the time when delivery of epirubicin was completed (Fig 3, left panels). The mean levels of α-hydroxybutyrate dehydrogenase, total CK and CK-MB were consistently higher in rats that received IP bolus than in rats that received micro pump infusion. Troponin-I was higher at 12 and 24 h after IP bolus than at the corresponding times after micro osmotic pump infusion (Fig 3, left bottom panel).

### Hematological evidence of a higher degree of bone marrow suppression by pump infusion than IP bolus injection of epirubicin

Despite initial elevation in the pump infusion group probably caused by surgical implantation of pump and in the intraperitoneal group probably due to peritoneal irritation, complete blood analysis with differential blood cell count showed that white blood cells (WBC) and neutrophils were lowered by epirubicin. Although the time courses for IP bolus and micro osmotic pump infusion were different, the levels were the same for both groups at 72 h after completion of administration of epirubicin (Fig 3, right panels). There were no significant changes in hemoglobin and platelets from the baseline caused by epirubicin.

### In vivo therapeutic efficacy of the two administration methods of epirubicin in a breast cancer mouse model

**Fig 4:**
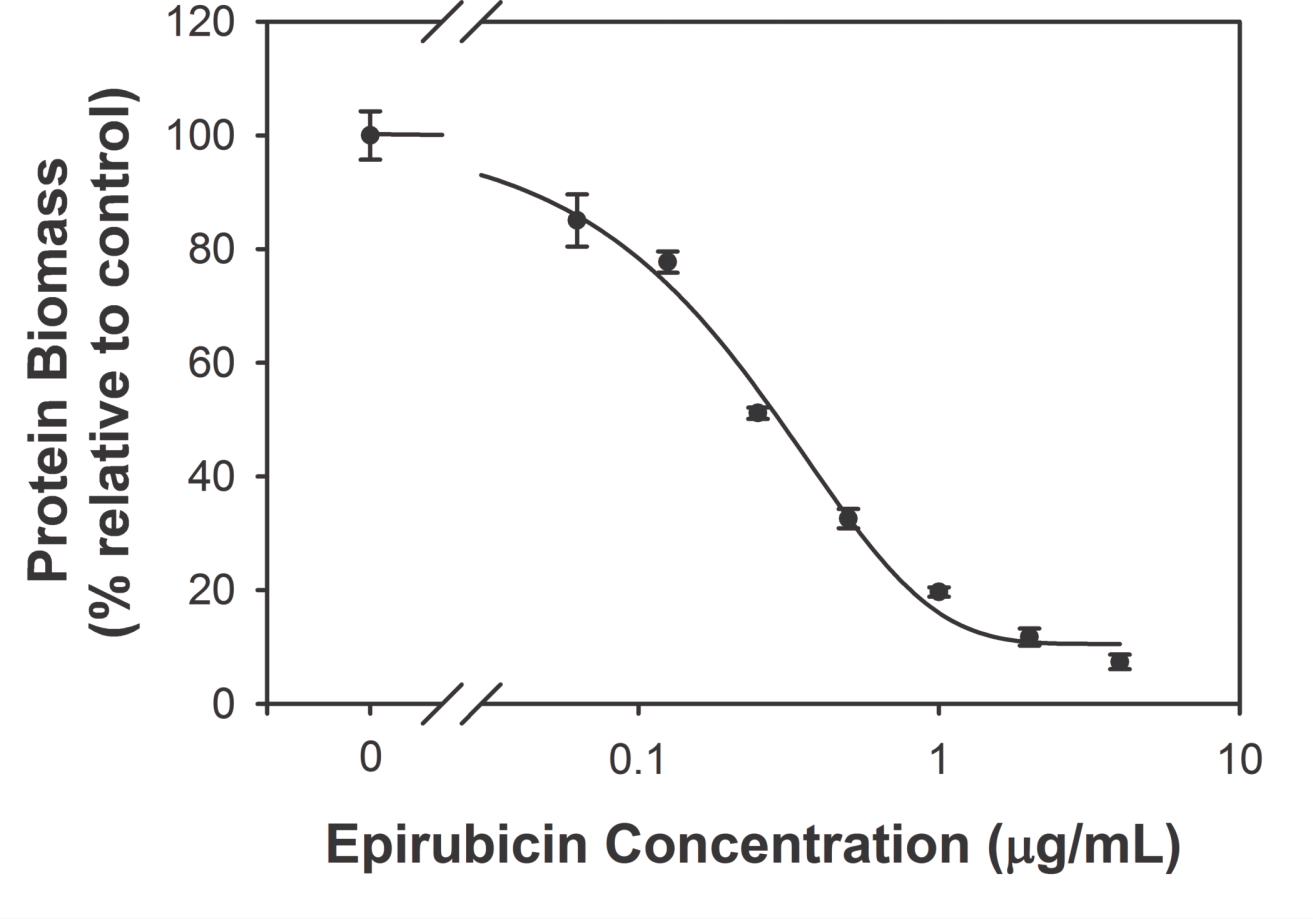
The log dose-response curve of the 4T1-luc cell line to epirubicin. The protein biomass relative to control was measured by the sulforhodamine B assay. A sigmoidal curve was fitted to the data. Error bars represent 95% confidence intervals.

The *in vitro* dose-response curve of the mouse breast cancer cell line 4T1-luc (see methods) was measured for epirubicin. Using the SRB assay to measure biomass of the cultured cells, the log-dose-response curve showed a 50% inhibitory concentration (IC_50_) of epirubicin of about 0.3 μg/mL (Fig 4).

**Fig 5:**
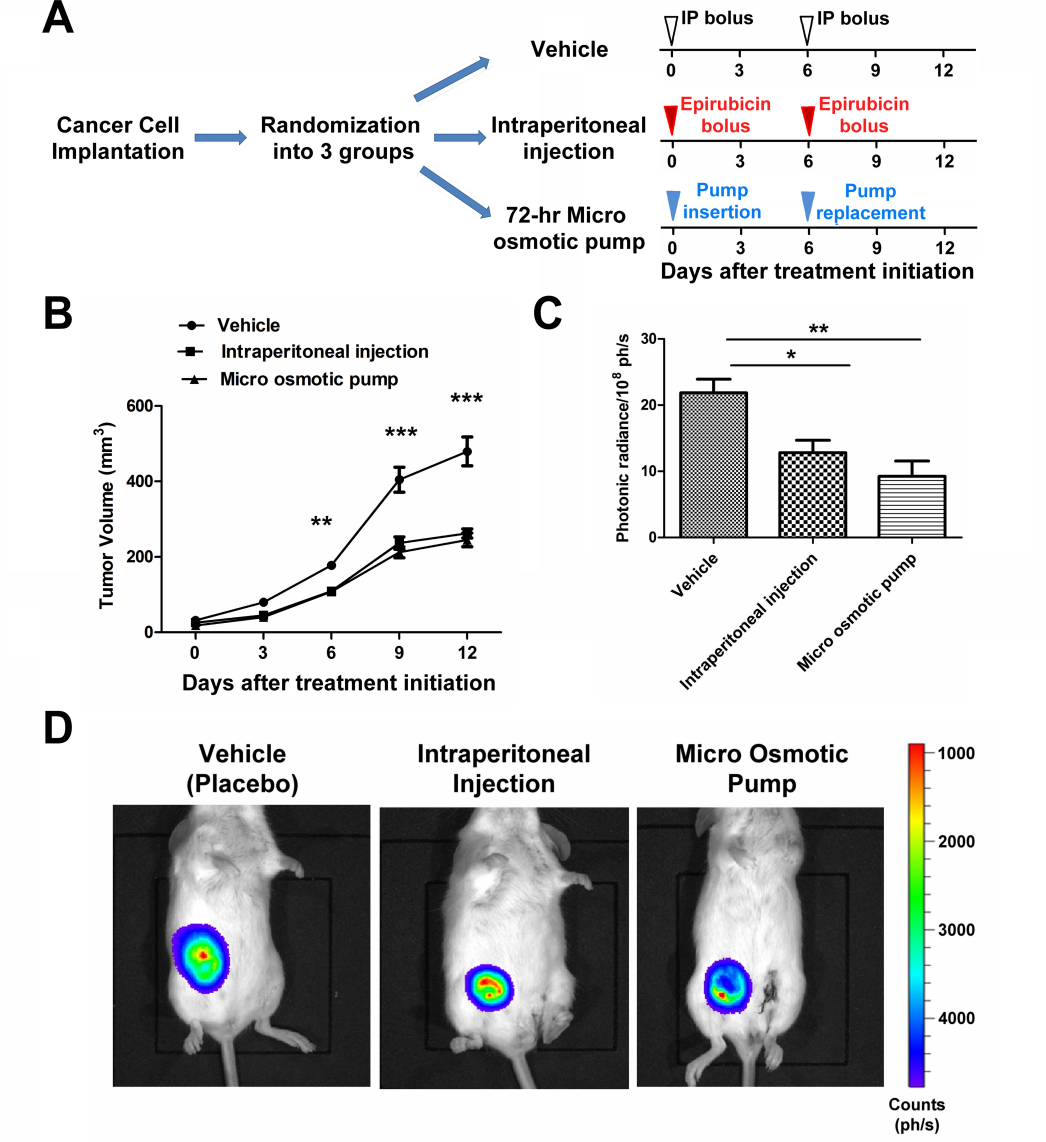
Equal *in vivo* antineoplastic efficacy of two methods of epirubicin administration. (A) Schematic representation of the experimental design and timing of data and sample collection from mice. (B) The tumor growth curves of orthotopic breast cancer allografts are shown for the three different groups of mice as labeled. Error bars represent standard errors; **, P<0.01; ***, P<0.001. (C) The photonic radiance from tumors was measured by bioluminescence imaging at 10 min post injection of D-luciferin. The error bars represent standard errors; *, P<0.05; **, P<0.01. (D) A representative comparison of bioluminescence intensity at 10 min in the three labeled groups of mice is shown. The color scale of the photon counts is labeled to the left of the images.

The orthotopic allograft breast cancer mouse model was used as described in Methods. The tumorigenic rate of 4T1-luc cells was 100%. The diameter of tumors reached ≥3 mm in 6 days after inoculation when the treatments began upon randomization into the 3 groups (Fig 5A). Mice from each group (blank group, epirubicin micro osmotic pump group, and intraperitoneal injection group) were examined for tumor growth both by caliper measurement of tumor size. The average tumor size was smaller in the epirubicin groups than in the blank group. There was no significant difference in tumor size between the IP bolus group and the pump group (Fig 5B). On Day 12, the tumor volumes were 262.34 ± 26.89 mm^3^ (mean ± standard deviation) and 244.95 ± 40.42 mm^3^ for the IP bolus group and the pump group respectively with a statistical power (1−β) of 0.874. Imaging of tumor bioluminescence using D-luciferin was also performed on the twelfth day after initiation of treatment. The photonic radiance measured at 10 min post injection of D-luciferin showed that both IP bolus of epirubicin and pump infusion of epirubicin inhibited tumor group to similar degrees. Again there was no significant difference in photonic radiance between the pump group and the IP bolus group (Fig 5C and 5D; P = 0.265 for IP vs pump; P = 0.004 for placebo vs pump; P = 0.012 for placebo vs IP; one-way ANOVA with post-hoc comparisons). Data measured at 5 min and 15 min after D-luciferin injection yield similar results (data not shown).

## Discussion

Our results clearly demonstrated that micro osmotic pump infusion of epirubicin has less cardiotoxic effects on rat myocardium as demonstrated both by histopathology and blood biomarkers of myocardial injury. The pharmacokinetic profiles show that slow infusion smooths the epirubicin plasma level and avoids the high peak plasma concentration seen in bolus administration. Therefore, our results in this report provides direct histopathological and plasma biomarker evidence of myocardial injury with pharmacokinetic correlation to demonstrate that avoidance of a high peak plasma concentration of epirubicin by slow infusion protects against myocardial injury compared with bolus administration of epirubicin.

Similar to what had been tried for doxorubicin [7], a 4-day infusion schedule for epirubicin was investigated for leukemia [13]. We previously studied a 48-hour infusion of epirubicin (70 mg/m^2^ in total) [14–16] with the goal to reduce the cardiotoxicity of epirubicin, and we found that continuous intravenous infusion of epirubicin using micro-pumps had less cardiotoxicity than intravenous bolus infusion of epirubicin at the same total dose of 70 mg/m^2^ [14, 17]. Since direct evidence by myocardial biopsy carries significant risk and burden to patients, we have sought experimental evidence in rodents. In our previous clinical observation [17], the pharmacokinetic analysis in humans was similar to our results in rats. The peak concentration of epirubicin in the pump infusion group was lower than bolus administration with the time of peak concentration shortly preceding the time of obvious myocardial injury. In this animal study, Alzet micro osmotic pumps were used; since these pumps were only available for 24-hour or 72-hour infusion and both 2-day and 4-day infusions have been used clinically before, we chose the 72-hour pumps in our experiments. Our results provide experimental evidence to support that the clinical practice of slow intravenous infusion of epirubicin over 2−4 days will decrease myocardial injury by avoiding high peak plasma concentrations of epirubicin.

Our data showed that pump infusion may prolong the duration of epirubicin-induced neutropenia, but the degree of neutropenia is similar for both bolus delivery and micro osmotic pump infusion at 72 h after completion of epirubicin delivery. In our previous clinical study [16], we did observe grade 4 neutropenia in 22.5% of patients who received epirubicin by micro-pump infusion but only in 8.8% of patients who received epirubicin by IV bolus injection. Therefore, our animal experimental data recapitulate our previous clinical observation.

A key question is whether the cardiotoxicity of epirubicin can be reduced without loss of therapeutic efficacy. Using an orthotopic allograft breast cancer mouse model, we did not find any significant difference in breast tumor inhibition between delivery by IP bolus and by IP micro osmotic pump infusion at a statistical power of 0.874. Therefore, lowering the peak plasma concentration of epirubicin by slow infusion does not reduce the antineoplastic efficacy of epirubicin.

In recent years, a popular method for cardioprotection from anthracyclines is to use cardioprotective agents (e.g., dexrazoxane) in addition to new formulations of anthracyclines (e.g., PEGylated liposomal doxorubicin). Although these approaches can reduce cardiac toxicity, they create significant financial burdens for patients in many parts of the world. In contrast, micro pump continuous infusion of epirubicin can be administered in an ambulatory setting; it is easy to implement and is readily acceptable to the patients.

A limitation of our study is that IP drug delivery is not the same as IV. Since cannulation of a large vein for continuous infusion and bolus tail vein injections are both technically challenging, we opted for IP. Our pharmacokinetic results should be interpreted with caution, keeping in mind that epirubicin does undergo first-pass metabolism in the liver [20]. Nevertheless, we can conclude that slow continuous infusion can avoid high peak plasma concentrations of epirubicin to offer cardioprotection shortly after infusion without compromising therapeutic efficacy. Other limitations of this study include the lack of data about multiple dosing using the different delivery methods and the lack of long term cardiotoxicity data. Further preclinical evaluation and prospective clinical study of this drug delivery method are warranted.
